# Comparing News Articles and Tweets About COVID-19 in Brazil: Sentiment Analysis and Topic Modeling Approach

**DOI:** 10.2196/24585

**Published:** 2021-02-10

**Authors:** Tiago de Melo, Carlos M S Figueiredo

**Affiliations:** 1 Intelligent Systems Laboratory Superior School of Technology Amazonas State University Manaus Brazil

**Keywords:** COVID-19, Twitter, infodemiology, news, sentiment analysis, social media, Brazil, monitoring, topic modeling, entity recognition, text analysis

## Abstract

**Background:**

The COVID-19 pandemic is severely affecting people worldwide. Currently, an important approach to understand this phenomenon and its impact on the lives of people consists of monitoring social networks and news on the internet.

**Objective:**

The purpose of this study is to present a methodology to capture the main subjects and themes under discussion in news media and social media and to apply this methodology to analyze the impact of the COVID-19 pandemic in Brazil.

**Methods:**

This work proposes a methodology based on topic modeling, namely entity recognition, and sentiment analysis of texts to compare Twitter posts and news, followed by visualization of the evolution and impact of the COVID-19 pandemic. We focused our analysis on Brazil, an important epicenter of the pandemic; therefore, we faced the challenge of addressing Brazilian Portuguese texts.

**Results:**

In this work, we collected and analyzed 18,413 articles from news media and 1,597,934 tweets posted by 1,299,084 users in Brazil. The results show that the proposed methodology improved the topic sentiment analysis over time, enabling better monitoring of internet media. Additionally, with this tool, we extracted some interesting insights about the evolution of the COVID-19 pandemic in Brazil. For instance, we found that Twitter presented similar topic coverage to news media; the main entities were similar, but they differed in theme distribution and entity diversity. Moreover, some aspects represented negative sentiment toward political themes in both media, and a high incidence of mentions of a specific drug denoted high political polarization during the pandemic.

**Conclusions:**

This study identified the main themes under discussion in both news and social media and how their sentiments evolved over time. It is possible to understand the major concerns of the public during the pandemic, and all the obtained information is thus useful for decision-making by authorities.

## Introduction

### Background

In December 2019, the outbreak of COVID-19 in China was reported [1]. Due to the rapid spread of SARS-CoV-2 worldwide, the World Health Organization declared a state of emergency. Recent research has confirmed that during the current pandemic, the number of infected people can double every 7 days, and each patient can spread the virus to 2.2 other people on average [2]. In Latin America, there were 937,974 cases of COVID-19 and 49,139 confirmed deaths up to May 31, 2020 [3]. In this region, Brazil is the country that is most affected by the disease. According to the abovementioned report [3], there were 465,166 cases and 27,878 deaths in Brazil.

In past pandemic outbreaks, information exchange was relatively slow. However, with the popularization of the internet, 3.7 billion people worldwide (approximately 49.7% of the world’s population) commonly use web-based information [4], and these people rely on two main sources of web-based data, namely news media web sites and social media. Using these media, people keep themselves informed about treatments, prevention, and cases, and they also participate in debates about the impact of the COVID-19 outbreak on their lives.

News media web sites are used to report crisis situations worldwide. The articles on these sites are written by journalists and subject matter experts; therefore, people trust these sources of data. However, these channels failed to keep pace with the spread of the outbreak of COVID-19 [5], and many news media channels incorrectly stated that either the pandemic would not affect countries other than China or the virus was less dangerous than influenza [6]. This coverage of the pandemic had repercussions after the spread of the disease became severe and global [7].

On the other hand, social media is a well-known channel for news and information in the timely media environment, with one in three people worldwide engaging in social media and two-thirds of people using the internet [8]. This is particularly true for health issues, with one-third of people reporting that social media are an important source of information [9]. However, recent studies have indicated that social media has also become an environment for misinformation on COVID-19 [10,11].

Currently, almost 70% of Brazilians use the internet, 90% of them access the web on a daily basis, and Brazil is the country in the western hemisphere whose residents spend most time on social media per day [12]. Thus, we envisioned that Brazil is a strategic country to study the impact of the COVID-19 pandemic through web-based media. We proposed to perform this task by applying an improved topic model and sentiment analysis methodology to news and social media compared to related work. The results of this study can help researchers understand what information about the pandemic is relevant and how people are reacting to it. Thus, this information can be useful for researchers and authorities to identify important aspects of the pandemic that can guide better action and communication policies toward the population.

### Prior Work

Traditional news media focus substantial interest on health issues, especially when a new disease emerges. A number of researchers have exploited the importance of understanding the depiction of health issues in the news media. For instance, Washer [13] investigated how severe acute respiratory syndrome (SARS) was depicted in newspapers in the United Kingdom. Dias et al [14] presented a study that analyzed the representations of mental health and its treatment and the impact of the 2008 economic crisis. Ribeiro et al [15] investigated how the Zika outbreak was reported in two major newspapers in Brazil. Liu et al [16] investigated the patterns of media-directed health communications as well as the role of the media in the COVID-19 crisis in China. Gozzi et al [17] investigated the media coverage and collective internet response to the COVID-19 pandemic in four countries: Italy, the United Kingdom, the United States, and Canada.

These related studies focused on how traditional news media react to health events and the characterization of their reports. Our work differs by focusing on the analysis of social media and comparing it with traditional news media, as we are interested in showing the impact of the COVID-19 pandemic on people’s lives.

The research community is also interested in correlating pandemic events with information shared by people on social networks, especially Twitter. Several examples show how useful information can be extracted from social media to help understand pandemic behavior but also to enable organizations to act to improve people’s quality of life. For instance, Chew and Eysenbach [18] presented the first study using Twitter data to evaluate the H1N1 influenza pandemic in 2009, showing that this social media platform disseminated news from credible sources but also shared users’ opinions and experiences. Comito et al [19] presented a study to evaluate the effectiveness of Twitter-based influenza as surveillance information. Ahmed et al [20] investigated the content shared by Twitter users during the Zika virus outbreak in 2016, and they showed that people's fears were intensified due to false news. More recently, Lwin et al [21] examined worldwide trends of several types of emotions and the narratives underlying those emotions during the COVID-19 pandemic. Abd-Alrazaq et al [22] presented a topic study of tweets in English, and Huang et al [23] analyzed the characteristics of suspected or laboratory-confirmed patients with COVID-19 who asked for help on social media; they found that it is possible to identify common patient characteristics in advance to accelerate emergency responses.

Although several previous studies have separately assessed news coverage and social media in pandemic events, only a few of them have compared news coverage with social media (in contrast to other disasters [24-28]). Particularly, Kim et al [29] investigated topic coverage and sentiment dynamics of two different media sources, Twitter and news publications, on the health issue of Ebola virus. The results reported in their paper indicate that Twitter and news media present two distinct points of view. In other work, Mondragon et al [30] presented a study on how Ebola virus was transformed from purely scientific knowledge to public thinking through media communication.

Our work follows a similar approach to that of [29] in that we use topic analysis and sentiment polarity on each data set. However, we have extended and improved the proposed methodology by generating the topic model from all data sets, aggregating them in meaningful themes, and analyzing sentiments from documents classified according to themes, which resulted in a better and more meaningful sentiment timeline.

### Goals

In this study, we describe a methodological approach to analyze the content of two main sources of web-based data to better understand the focus of each channel in disseminating information on COVID-19. Recent work in the literature (eg, [18,19]) has presented methodologies focused on social media and news comparisons based on topic models [31] and sentiment analysis [32]. We have contributed to the literature by extending these methodologies in addition to introducing specific analysis to understand the COVID-19 pandemic in Brazil. To the best of our knowledge, this is the first study to compare news and social media data in Portuguese.

The three main research questions that we are addressing in this study are:

- RQ1: Does social media cover similar categories and types of topics to traditional news media about the COVID-19 pandemic?
- RQ2: Do news web sites and social media mention the same types of entities?
- RQ3: Are there differences in the sentiments of Twitter posts and news articles? Does the degree of sentiments change over time?

To answer these questions, we collected and analyzed data from the main news media web site from Brazil, namely Universo Online (UOL), and Twitter. Twitter is a very popular social media platform worldwide, and UOL is a very popular portal for news in Brazil. We proposed the generation of topic models for each data collection, their grouping in themes for sentiment analysis, the observation of theme-sentiment evolution on a time scale, and the extraction of named entities. One challenging aspect of this research is the adaptation of the proposed methods to the Brazilian Portuguese language; therefore, we adopted some tools and developed specific trained models. By comparing all the features extracted from news and social media data sets, we present some perceptions on how the COVID-19 pandemic is affecting Brazil.

## Methods

### Data Collection

We collected news articles and tweets related to COVID-19 in the Portuguese language from January to May 2020. To collect the tweets, we used the TwitterScraper Python library [33] with the option *--lang* to retrieve tweets only in Portuguese. The metadata of a tweet contains a location entry; however, we noted that very few users fill in this field, and many of those users fill in nonstandard labels. Although Brazil is not the only country in which Portuguese is spoken, it represents 75% of the world’s speakers, and upon manually checking the tweets that contained the user’s location, we observed that only 4% were from people who spoke Portuguese and were not in Brazil. Thus, we consider that these data statistically represent this country. We also filtered the tweets with the following set of most frequently appearing keywords obtained from Google Trends for COVID-19–related topics: *azitromicina* (azithromycin), *cloroquina* (chloroquine), *comorbidade* (comorbidity), *corona*, *coronavirus*, *covid*, *covid19*, *covid-19*, dis*tanciamento social* (social distancing), *ivermectina* (ivermectin), *lockdown*, *hidroxicloroquina* (hydroxychloroquine), *pandemia* (pandemic), *quarentena* (quarantine), and *tamiflu*. This search for keywords was executed at the beginning of March 2020. The final Twitter collection did not contain any retweets, and it contained 1,597,934 tweets posted by 1,299,084 users.

Regarding news collection, we gathered all the articles published in the COVID-19 section from the UOL portal. We chose UOL because this portal is responsible for publishing the *Folha de São Paulo*, which is the leading Brazilian daily newspaper by circulation [34]. In this collection, we gathered all web pages related to COVID-19; therefore, we did not need to use a set of keywords. The final news collection contained 18,413 articles.

### Characterization of the Collected Data

**Figure 1:**
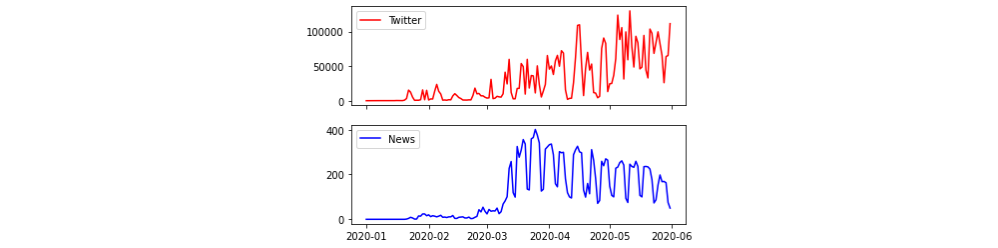
Distribution of tweets and news reports by day.

To better understand the collected data, we evaluated the statistics of the number of tokens published in each data source over time, where a token is an individual occurrence of a linguistic unit in speech or writing. The monthly distributions of the total number and percentage of tokens from both data sets are described in Table 1. One major difference between tweets and news articles is that the news presented a sharp increase in the number of tokens that decreased in May, whereas a persistent increase occurred on Twitter during the entire period of time. These findings indicate that Twitter users remained increasingly interested in the COVID-19 pandemic, while the news media began to lose interest in the month of May. This can also be observed in Figure 1, which shows the distributions of the collected data by day over five months. Additionally, this figure shows that the number of posts sharply increased at the end of March, when the first death from COVID-19 was announced in Brazil.

**Table 1:** Monthly statistics of tokens in news articles and tweets.

Tokens		Month, n (%)				
		January	February	March	April	May
**News articles**						
	Unique tokens (n=134,845)	4149 (3.07)	10,093 (7.48)	38,300 (28.40)	43,327 (32.13)	38,976 (28.90)
	Total tokens (n= 2,616,002)	14,550 (0.55)	62,008 (2.37)	792,175 (30.28)	953,441 (36.44)	793,828 (30.34)
**Tweets**						
	Unique tokens (n=407,406)	16,100 (3.95)	29,511 (7.24)	84,713 (20.79)	122,169 (29.98)	154,913 (38.02)
	Total tokens (n=14,155,346)	106,619 (0.75)	284,012 (2.00)	2,191,226 (15.48)	4,420,658 (31.23)	7,152,831 (50.53)

**Figure 2:**
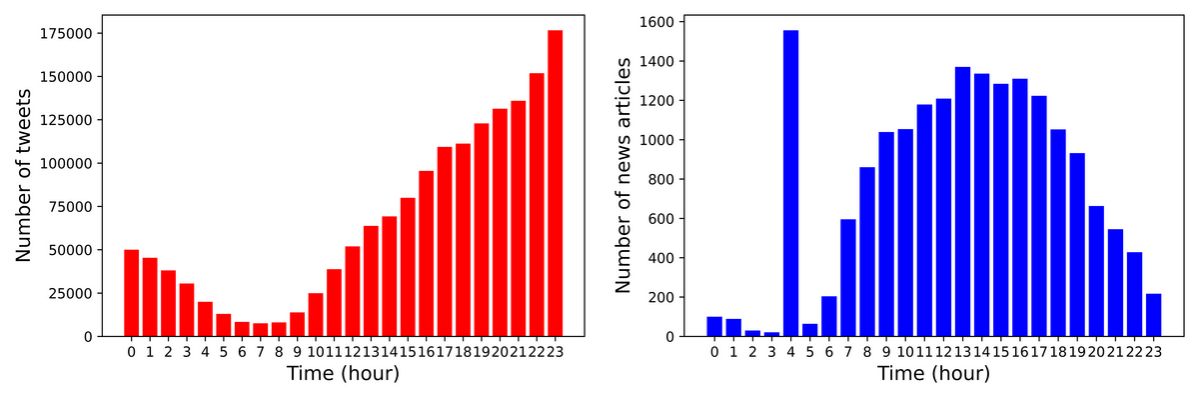
The 24-hour temporal distributions of posted tweets and news articles.

We also noted that the variation in the density of news and tweets over time (Figure 2) shows the 24-hour temporal distributions of news and tweets. The highest rates of tweeting occurred at night, while the highest rates of news posting occurred between noon and 6 PM. Interestingly, we verified that a peak in the news data source appeared at 4 AM on different days. This is probably due to an automated action to publish news for that morning.

**Figure 3:**
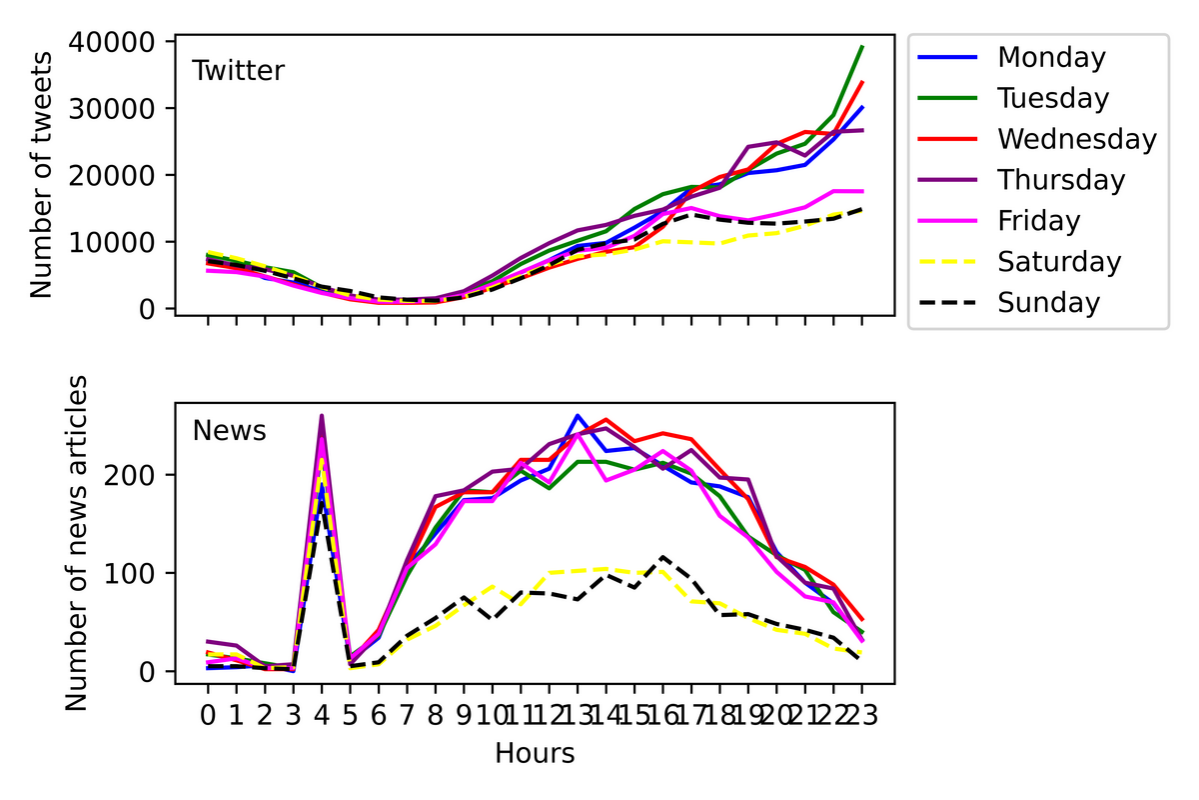
The 24-hour temporal distribution of posts on Twitter and Universo Online.

Although the distribution trend is relatively consistent on each day of the week, the activity was significantly different between work periods and holidays. As shown in Figure 3, during work periods, the number of tweets and news publications was significantly higher. The daily activity during holidays was quite different from that during work periods. One of the main differences observed between data sources is that Twitter users posted on the weekend until early afternoon at the same pace as during work periods, while news articles were posted at a much slower pace during holiday periods than during work periods.

### Data Preprocessing

The collected data contained a large amount of noise that needed to be filtered out before further analysis. First, we tokenized the text, and then we adopted the following steps to normalize the texts:

1. Lowercase: All tokens were converted to lowercase. By doing this, identical tokens were merged and the dimensionality of the text was reduced.
2. URL removal: People post URLs with text to provide supporting information about the text. These URL links became noisy data during the analysis. All URL links in the texts were replaced by a space.
3. Username: Some Twitter usernames in texts start with the symbol @ and are used to tag other users. In our investigation, we were focusing on COVID-19 and not on any targeted person; therefore, we replaced all usernames with white spaces. This step was applied only to tweets.
4. Punctuation: We removed all the punctuation symbols from the collected data because they did not contribute to our evaluation.
5. Stop words: Stop words refer to the most common words used in text. We eliminated the Portuguese stop words that contributed less to our evaluation. We used a list of Portuguese stop words provided by the Natural Language Toolkit framework.
6. White spaces: We removed all the extra white spaces between tokens or at the end of lines or paragraphs of the text.

In addition to the above steps, we used lemmatization and stemming in the preprocessing of the text. However, the results were not satisfactory because there are few tools with these functions in the Portuguese language, and these tools present results with low accuracy.

### Topic Modeling and Topic Similarity

Topic models are particularly useful because they enable the inference of structure from a large data collection without the need for extensive manual interventions [35]. In the sentiment analysis domain, one of the best-known techniques to discover topics is latent Dirichlet allocation (LDA) [36]. LDA is a statistical topic model with the purpose of automatically identifying groups of related terms that approximate to real-word topics. In our research, we used LDA to uncover the main discussion topics and their trends over time.

**Figure 4:**
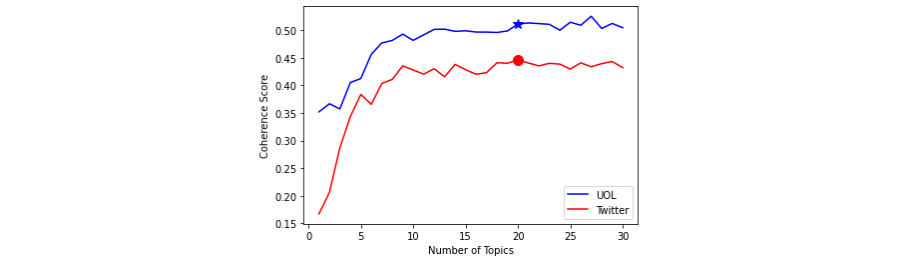
Coherence scores for the latent Dirichlet allocation. UOL: Universo Online.

LDA requires the user to specify the number of topics, where this parameter provides control over the granularity of the discovered topics. A larger number of topics will produce more detailed topics (finer-grained), while a smaller number of topics will produce more general topics (coarser-grained). Therefore, there is no single value of the number of topics that is appropriate in all domains and types of problems. To discover the most appropriate number of topics, we performed several different LDA experiments, varying the number of topics from 1 to 30 for both data sources. As illustrated in Figure 4, the coherence score increases steadily and quickly at the beginning, but it becomes stable at the score of 10 for both data sources. With the goal to capture broad topic trends in both data collections while keeping them distinct from each other, we set the number of topics to 20. Our final model generated 20 topics using the MALLET (Machine Learning for Language Toolkit) implementation of LDA [37] with the default parameters, and the coherence scores were 0.511 and 0.446 for news media and Twitter, respectively. The lowest values of the score obtained for Twitter are in line with previous results reported by [31,38,39], because LDA may not necessarily perform well when handling short texts. Despite this shortcoming, we still extracted a set of representative topics about COVID-19 from Twitter.

After topic discovery, we manually categorized the topics in themes based on the first 10 words, as these terms are ranked by their probability of appearance. The topics were categorized in the following themes: Confirmed Cases, Economic Influences, Entertainment, Medical Supplies, Medical Treatment and Research, Political, Prevention and Control, and Stories. Table 2 presents a short description of each theme. These themes are commonly used in the literature [16,22,40] to aggregate topics discussing similar subjects.

**Table 2:** Descriptions of the considered themes in this work.

Theme	Description
Confirmed Cases	Mentions of confirmed cases of COVID-19, such as updated numbers of cases and mortalities
Economic Influences	The influence of COVID-19 on the economy and society, such as the large number of unemployed people
Entertainment	Cultural events, sports, or food, such as the interruption of soccer championships
Medical Supplies	The medical supply situation in Brazil, such as the lack of respirators and use of masks
Medical Treatment and Research	Mentions of medical treatment and research combating COVID-19, such as the use of hydroxychloroquine
Political	Mentions of politicians and public officials and their responsibility
Prevention and Control	Different aspects of prevention and control procedures, such as social isolation and lockdowns in cities
Stories	Stories from people in Brazil who became ill or about the impact of COVID-19 on people’s lives

Once we obtained the topics and themes, we assessed their similarity to understand if Twitter and traditional news media cover similar categories and types of topics related to COVID-19. To achieve this, we adopted the popular cosine similarity, which is the angle between the representation of two topics, as a measure to report the similarity among topics:


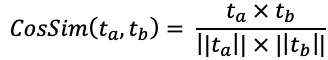


where *t_a_* and *t_b_* are the vector representations of topics *a* and *b*, respectively. The range of similarity is between 0 and 1, as all vector values are positive, with 1 implying total similarity.

### Calculation of Topic-Based Sentiment Scores

For the sentiment analysis, we identified the polarity of the opinion or emotion expressed in the texts. One challenge we faced was the lack of robust language resources to support sentiment analysis for the Portuguese language [41]. This is due to the lack of advanced tools for Portuguese. Some studies [32,42,43] showed that methods that translate data set texts to English for sentiment analysis perform better than versions in Portuguese. In fact, this approach has become very common in research involving multilingual texts, as machine translation systems are presenting a good level of maturity [44]. Thus, we adopted this approach in this work and translated all original texts to English using the googletrans tool, a free Google Translate application programming interface [45], which is also evaluated in [44] and used in [46].

After the translation process. we used the VADER (Valence Aware Dictionary and Sentiment Reasoner) tool [47] to calculate the degree of positivity or negativity of the texts. VADER is a lexicon and rule-based sentiment analysis tool that focuses on sentiments expressed in social media. It can capture sentiment intensity based on grammatical and syntactical conventions. It performs well on social media platforms such as Twitter. Evaluations in [47] also show competitive performance on conventional text data sets. Sentiments are assigned a value on a scale of –1.0 to 1.0, the most negative and most positive polarities, where 0.0 represents neutral.

Table 3 presents some examples. It can be seen that the translation tool captures expressions and social typos well (eg, *vc* = *você* = you) while preserving entity names.

**Table 3:** Examples of text translation and sentiment analysis.

Portuguese text	Translated text	VADER^a^ sentiment
*pq é que pessoas doentes insistem em espirrar para cima dos outros? nc ouviram falar de distanciamento social quando estão doentes?*	why are sick people insisting on splashing on others? haven’t you heard of social distance when you’re sick?	–0.8
*Pode o que vc quiser, seu corpo, suas regras.* *Fim.*	Do what you want, your body, your rules. End.	0.0
*ministro Paulo Guedes diz que vamos recuperar e em ”V”!!! Isso significa, rapidamente! Eu confio nele!*	Minister Paulo Guedes says that we will recover and in ”V” !!! That means, quickly! I trust him!	0.8

^a^VADER: Valence Aware Dictionary and Sentiment Reasoner

### Named Entity Recognition and Network

Named entity recognition (NER) is particularly useful for identifying which terms in a text are mentions of entities in the real world and classifying them according to a set of categories. Although NER is not a new research field, it is not an easy task. The reasons for this are multifold. First, there is much work targeting English text, but studies focused on Portuguese text are still scarce [48]. Therefore, further work is still needed for several languages, such as Portuguese, due to their complex structures and relatively scarce language processing tools and data sets. Secondly, the category of a named entity is highly dependent on textual semantics and its surrounding context. The extraction of named entities from Twitter is an even more challenging task because tweets are short and are therefore more difficult to interpret compared to longer texts. In addition, short texts have many linguistic variations, and they tend to be less grammatically correct than news articles. Moreover, there are many definitions of named entity and evaluation criteria, which introduces evaluation complications [49]. Finally, we could not find a NER system that was designed to recognize the entities that we were interested in for this study.

Considering that the current state-of-the-art NER systems are based on neural architectures, we decided to use the spaCy2 library, which is based on the hierarchical attention network proposed in [50] and enables the creation of news models. The pretrained model for named entity recognition in Portuguese provided by spaCy recognizes the following entities: location, organization, person, and miscellaneous. As our goal is to identify entities in tweets and news articles related to COVID-19, we created our own model using the spaCy library. Our model is able to recognize the following entities: Person (PER), Organization (ORG), Disease (DIS), Symptoms (SYMP), and Drugs (DRUG). We chose these categories of entities because they are essential during a pandemic crisis.

**Figure 5:**
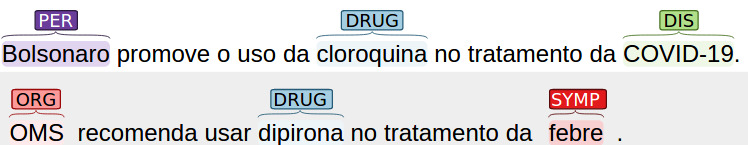
Sample outcome of the trained NER model.

We trained a new blank spaCy Portuguese language model; the initial model had no trained entities. An important issue in generating NER models is the effort involved in obtaining training data. To address this issue, we adopted a semisupervised approach to create training data that is better explained as follows. After training data generation, we then shuffled and looped over the training data. For each instance, the model was updated by calling the update function, which steps through all the words of each sentence. At each word, the update function makes a prediction. It then consults the golden standards to determine whether the prediction is right. If it is wrong, the update function adjusts its weights so that the correct action will score higher next time. Our model was built using 100 iterations with a dropout rate of 0.2. Once trained, our NER model was saved, and it can be used to recognize named entities in previously unseen tweets and news. Figure 5 illustrates an output example of our NER model, where *dipirona* (dipyrone) is a type of drug that was not used in training.

Regarding the training data set, our strategy consisted of using a list of keywords for each entity regarding COVID-19 as a set of seeds. The algorithm in Textbox 1 describes this semi-supervised strategy. The algorithm takes two inputs. The first input is a set of pairs *〈e, k〉 ∈ P,* where *e* is an entity and *k* is a keyword. *〈DRUG,tamiflu〉* and *〈DIS,COVID-19〉* are examples of pairs. The second input is a set *S* of unlabeled sentences. We used the sentences from our data collection. Our algorithm returns as output a training data composed of pairs *〈s,L〉*, where *s* is a sentence and *L* is a list of pairs *〈e, k〉*. Each of these pairs represents that sentence *s* contains one or more keywords *k ∈ S* associated with some entity *e ∈ E*.

The algorithm iterates through the set of sentences *s ∈ S* (lines 4-12), attempting to match any of the sentence terms with some *k ∈ P* (line 7). If there is an occurrence of *k* with any term of the sentence s, then the pair *〈e, k〉* is added to the list *L* (Line 8). After all the pairs belonging to *P* have been processed, a training pair *〈s,L〉* is added to *T* in line 11. If the keyword *k* does not match any term in sentence *s*, this sentence *s* is simply discarded. Note that in this case, the set *L* remains empty. After all the sentences *s ∈ S* are processed, the algorithm outputs the training set *T* in line 13.

The training set generated by Algorithm 1 involves only a small degree of supervision, such as a set of keywords for each target entity, to start the learning process. To represent each type of text, we generated distinct training sets for news media and Twitter.

**Semisupervised learning strategy.**

1 **let** *E* be the set of entities;

2 **let** *K* be a set of keywords about COVID-19;

Input: A set P = {〈e, k⟩ | e ∈ E and k ∈ K}

**Input:** A set *S* of unlabeled sentences

**Output:** A set of training pairs {〈*s*,L〉 |*s ∈ S* and *L* is a list of *p ∈ P*}

3     *T ←∅*;

4    **foreach** *s ∈ S* **do**

5       *L←∅*;

6       **foreach** *e, k ∈ P* **do**

7          **if** *k ∈ {s}* **then**

8             *L ←L ∪ {〈e, k〉}*;

9          **end**

10       **end**

11       *T←T ∪ {〈s,L〉}*;

12    **end**

13    **return** *T*

## Results

### Overall Topic Distribution

Topics were analyzed for UOL and Twitter data sets according to the methods described in the previous section. Afterward, we organized the topics in themes as described in Table II. Topics and themes for UOL and Twitter are shown in Table 4 and Table 5, respectively. The original words in Portuguese are shown in brackets. The topic terms appear in decreasing order of density distribution. These tables show that the topic terms capture different meanings from both UOL and Twitter posts, and a diversity of themes was represented.

**Table 4:** Topics and themes for Universo Online.

ID	Topic	Theme
1	people (*pessoas*), mask (*máscara*), city (*cidade*), food (*alimentos*), products (*produtos*), employees (*funcionários*), local (*local*), alcohol (*álcool*), residents (*moradores*), image (*imagem*)	Prevention and Control
2	people (*gente*), do (*fazer*), stay (*ficar*), people (*pessoas*), time (*tempo*), account (*conta*), moment (*momento*), situation (*situação*), work (*trabalho*), folks (*pessoal*)	Stories
3	president (*presidente*), bolsonaro (*bolsonaro*), minister (*ministro*), state (*disse*), stated (*afirmou*), health (*saúde*), isolation (*isolamento*), interview (*entrevista*), party (*partido*), social (*social*)	Political
4	cases (*casos*), number (*número*), deaths (*mortes*), confirmed (*confirmados*), data (*dados*), total (*total*), bigger (*maior*), disease (*doença*), people (*pessoas*), deaths (*óbitos*)	Confirmed Cases
5	tests (*testes*), patients (*pacientes*), study (*estudo*), treatment (*tratamento*), research (*pesquisa*), vaccine (*vacina*), virus (*vírus*), researchers (*pesquisadores*), pain (*dor*), disease (*doença*), results (*resultados)*	Medical Treatment and Research
6	people (*pessoas*), virus (*vírus*), risk (*risco*), health (*saúde*), disease (*doença*), can (*podem*), diseases (*doenças*), avoid (*evitar*), population (*população*), seniors (*idosos*)	Prevention and Control
7	president (*presidente*), government (*governo*), states (*estados*), state (*estado*), minister (*ministro*), crisis (*crise*), pandemic (*pandemia*), congress (*congresso*), project (*projeto*), senate (*senado*)	Political
8	measures (*medidas*), isolation (*isolamento*), social (*social*), people (*pessoas*), state (*estado*), city (*cidade*), activities (*atividades*), capital (*capital*), cities (*cidades*), measure (*medida*)	Prevention and Control
9	can (*podem*), data (*dados*), information (*informação*), classes (*aulas*), access (*acesso*), possible (*possível*), do (*fazer*), work (*trabalho*), pandemic (*pandemia*), form (*forma*)	Stories
10	disease (*doença*), symptoms (*sintomas*), hospital positive (*positivo*), result (*resultado*), state (*disse*), death (*morte*), exams (*exames*), doctor (*médico*), covid (*covid*)	Medical Treatment and Research
11	state (*disse*), announced (*anunciou*), week (*semana*), pandemic (*pandemia*), march (*março*), communication (*comunicado*), events (*eventos*), april (*abril*), since (*partir*), june (*junho*)	Political
12	soccer (*futebol*), pandemic (*pandemia*), championship (*campeonato*), clubs (*clubes*), players (*jogadores*), season (*temporada*), athletes (*atletas*), games (*jogos*), return (*retorno*), english (*inglês*)	Entertainment
13	countries (*países*), state (*disse*), authorities (*autoridades*), world (*mundo*), people (*pessoas*), measures (*medidas*), worldwide (*mundial*), organization (*organização*), pandemic (*pandemia*), confinement (*confinamento*)	Political
14	economy (*economia*), companies (*empresas*), crisis (*crise*), fall (*queda*), market (*mercado*), sector (*setor*), pandemic (*pandemia*), bigger (*maior*), production (*produção*), impact (*impacto*)	Economic Influences
15	pandemic (*pandemia*), world (*mundo*), people (*pessoas*), moment (*momento*), big (*grande*), crisis (*crise*), population (*população*), form (*forma*), social (*social*), society (*sociedade*)	Stories
16	health (*saúde*), professionals (*profissionais*), patients (*pacientes*), hospitals (*hospitais*), beds (*leitos*), state (*estado*), doctors (*médicos*), attendance (*atendimento*), hospital (hospital), equipment (*equipamentos*)	Medical Supplies
17	decision (*decisão*), police (*polícia*), general (*geral*), public (*público*), ministry (*ministro*), request (*pedido*), state (*estado*), safety (*segurança*), public (*público*), measures (*medidas*)	Political
18	workers (*trabalhadores*), work (*trabalho*), government (*governo*), companies (*empresas*), payment (*pagamento*), income (*renda*), value (*valor*), caixa measure (*medida*), money (*dinheiro*)	Economic Influences
19	social nets (*redes*), video (*vídeo*), publication (*publicação*), instagram (*instagram*), twitter (*twitter*), wrote (*escreveu*), shared (*compartilhada*), quarantine (*quarentena*), world (*mundo*)	Stories
20	masks (*máscaras*), protection (*proteção*), coronavirus (*coronavirus*), passengers (*passageiros*), american (*americano*), local cases (*casos*), week (*semana*), final (*final*), transmission (*transmissão*)	Prevention and control

**Table 5:** Topics and themes for Twitter.

ID	Topic	Theme
1	president (*presidente*), bolsonaro (*bolsonaro*), minister (*ministro*), governors (*governadores*), sir (*senhor*), mayors (*prefeitos*), jairbolsonaro (*jairbolsonaro*), blame (*culpa*), mandetta (*mandetta*), meeting (*reunião*)	Political
2	instagram (*instagram*), igshid (*igshid*), covid (*covid*), twitter (*twitter*), mask (*máscara*), masks (*mascaras*), stay (*fique*), important (*importante*), prevention (*prevenção*), attention (*atenção*)	Medical Supplies
3	deaths (*mortes*), number (*número*), dead (*morte*), bigger (*maior*), covid (*covid*), numbers (*números*), countries (*países*), infected (*infectados*), weeks (*semanas*), months (*meses*)	Confirmed Cases
4	cases (*casos*), state (*estado*), confirmed (*confirmados*), tests (*testes*), city (*cidade*), twitter (*twitter*), deaths (*óbitos*), new (*novos*), coronavirus (*coronavirus*), total (*total*)	Confirmed Cases
5	health (*saúde*), hospitals (*hospitais*), combat (*combate*), professionals (*profissionais*), measures (*medidas*), public (*público*), actions (*ações*), beds (*leitos*), campaign (*campanha*), system (*sistema*)	Medical Supplies
6	twitter (*twitter*), pandemic (*pandemia*), covid (*covid*), lives (*vidas*), work (*trabalho*), moment (*momento*), video (*video*), congratulations (*parabéns*), big (*grande*), save (*salvar)*	Stories
7	people (*gente*), quarantine (*quarentena*), doing (*fazendo*), do (*fazer*), stay (*ficar*), friends (*amigos*), really (*sério*), damn (*porra*), finish (*acabar*), seeing (*vendo*)	Stories
8	pandemic (*pandemia*), time (*tempo*), quarantine (*quarentena*), things (*coisas*), moment (*momento*), time (*tempos*), difficult (*difícil*), do (*fazer*), pass (*passar*), expect (*espero*)	Stories
9	pandemic (*pandemia*), world (*mundo*), economy (*economia*), worldwide (*mundial*), general (*geral*), war (*guerra*), finish (*acabar*), ended (*acabou*), history (*história*), can (*podem*)	Economic Influences
10	true (*verdade*), policy (*política*), press (*imprensa*), left (*esquerda*), tell (*dizer*), state (*estado*), political (*político*), said (*falou*), media (*mídia*), shame (*vergonha*)	Political
11	people (*pessoas*), risk (*risco*), lack (*falta*), covid (*covid*), group (*grupo*), dying (*morrendo*), cause (*causa*), can (*podem*), died (*morreram*), diseases (*doenças*)	Confirmed Cases
12	social isolation (*isolamento*), detachment (*distanciamento*), measures (*medidas*), keep (*manter*), governor (*governador*), required (*necessário*), need (*necessidade*), services (*serviços*), commerce (*comércio*)	Prevention and Control
13	government (*governo*), population (*população*), combat (*combate*), money (*dinheiro*), pandemic (*pandemia*), fight (*combater*), want (*querem*), assist (*ajudar*), federal (*federal*), help (*ajuda*)	Political
14	death (*morte*), covid (*covid*), person (*pessoa*), disease (*doença*), positive (*positivo*), symptoms (*sintomas*), hospital died (*morreu*), week (*semana*), result (*resultado*)	Confirmed Cases
15	do (*fazer*), take (*tomar*), need (*precisa*), stay (*ficar*), die (*morrer*), work (*trabalhar*), want (*quero*), take (*pegar*), back (*voltar*), know (*saber*)	Economic Influences
16	corona (*corona*), virus (*vírus*), thing (*coisa*), speak (*falar*), state (*disse*), speaking (*falando*), cause (*causa*), buy (*comprar*), account (*voltar*), looks (*parece*)	Stories
17	coronavirus (*coronavirus*), twitter (*twitter*), covid (*covid*), vaccine (*vacina*), news (*notícias*), health (*saúde*), research (*pesquisa*), globo (*globo*), coronavirus (*coronavirus*)	Medical Treatment and Research
18	situation (*situação*), form (*forma*), problem (*problema*), exist (*existe*), best (*melhor*), done (*feito*), question (*questão*), possible (*possível*), looks (*parece*), example (*exemplo*)	Stories
19	pandemic (*pandemia*), crisis (*crise*), twitter (*twitter*), coronavirus (*coronavirus*), account (*conta*), companies (*empresas*), soccer (*futebol*), company (*empresa*), big (*grandes*), activities (*atividades*)	Entertainment
20	chloroquine (*cloroquina*), treatment (*tratamento*), patients (*pacientes*), doctor (*médicos*), medicine (*remédio*), study (*estudo*), doctors (*médicos*), medicine (*medicamento*), effects (*efeitos*), studies (*estudos*)	Medical Treatment and Research

**Figure 6:**
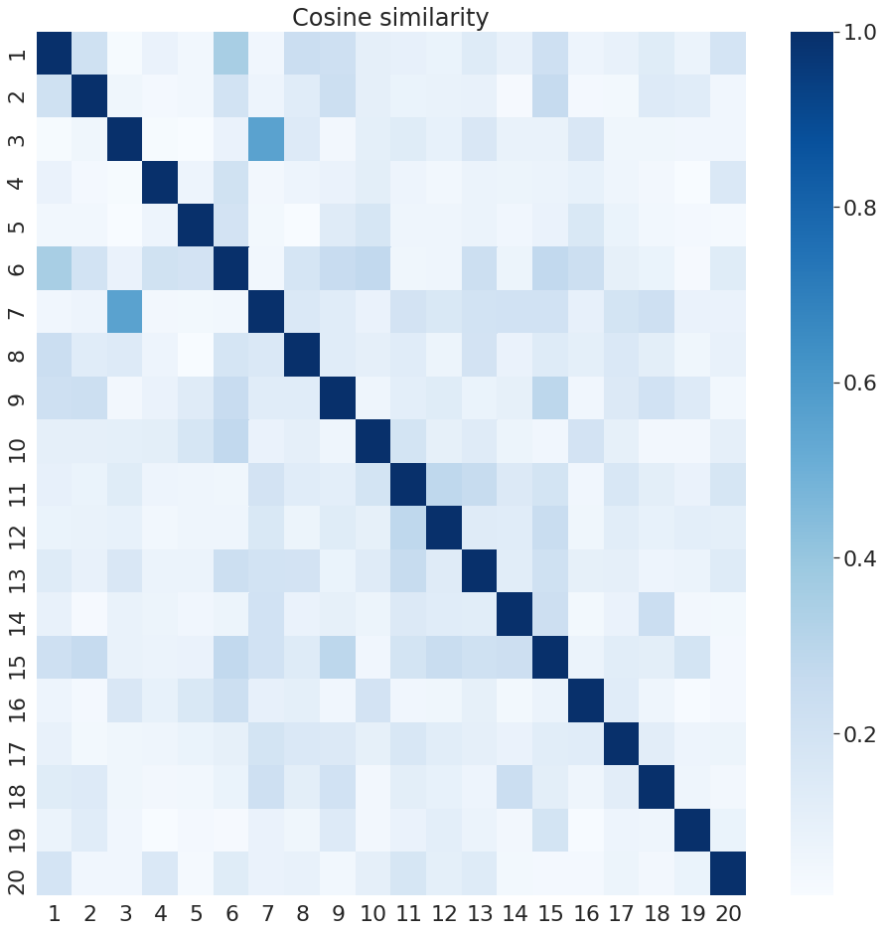
Similarity among Universo Online topics.

**Figure 7:**
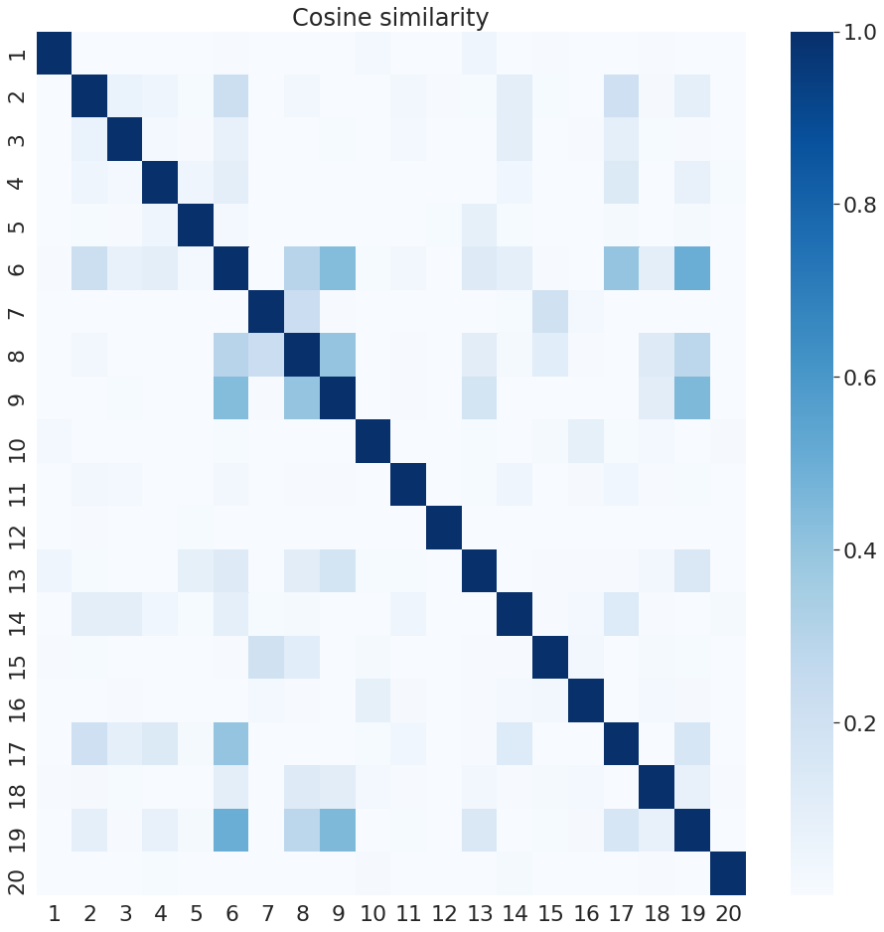
Similarity among Twitter topics.

Figure 6 and Figure 7 present the cosine similarities among the achieved topics of the UOL and Twitter collections, respectively. The goal was to observe the subject coverage obtained with these topics. We can note that the UOL news presents only two topics, which are very correlated (eg, 3 and 7 in Table 4 have many terms in common), while many other topics present lower correlations. This indicates that UOL topics cover more diverse subjects. Twitter, in contrast, presents more topics with stronger correlations (eg, 6, 9, 17, and 19) but also presents more topics with very low similarities. This is due to the fact that UOL documents are both formally written and longer than Twitter documents (which have a character limit). Thus, its documents naturally relate different subjects, and common terms can be found among different posts. Additionally, on social media, it is common for a few subjects to be concentrating the attention of the users, while people also talk about aleatory things.

**Figure 8:**
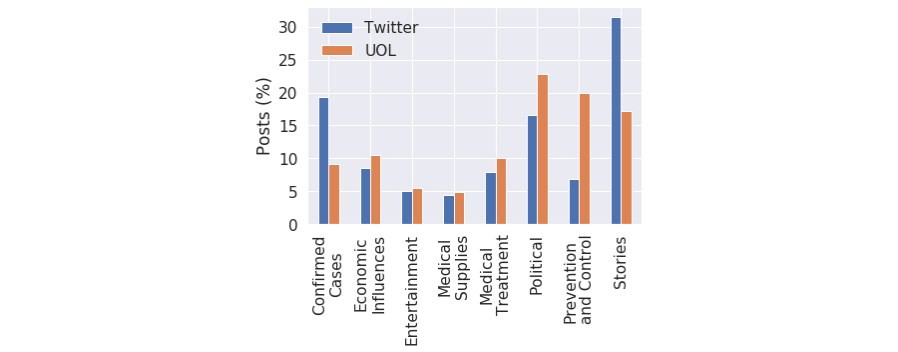
Distribution of themes. UOL: Universo Online.

The theme distributions between the UOL and Twitter collections are compared in Figure 8. In the UOL media, we can see that the preferred subjects are Politics, Prevention and Control, and Stories. One theme that was less common than expected is Confirmed Cases; however, this can be explained by the fact that this information was concentrated on fixed dashboards rather than in new documents. On Twitter, people posted frequently about the impact of COVID-19 on their lives (Stories), followed by concerns about Confirmed Cases and Political subjects, especially among supporters and critics of Brazil’s president.

### Entity Distribution and Network

**Figure 9:**
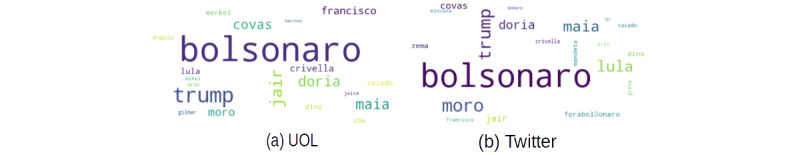
Word clouds showing the most frequent entity mentions in the Persons category: (a) Universo Online; (b) Twitter.

**Figure 10:**
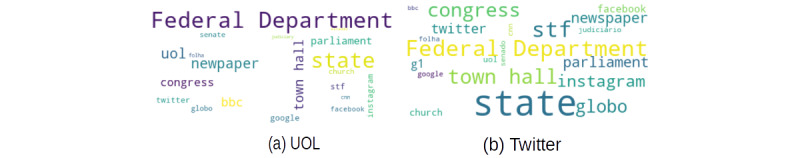
Word clouds showing the most frequent entity mentions in the Organizations category: (a) Universo Online; (b) Twitter.

**Figure 11:**
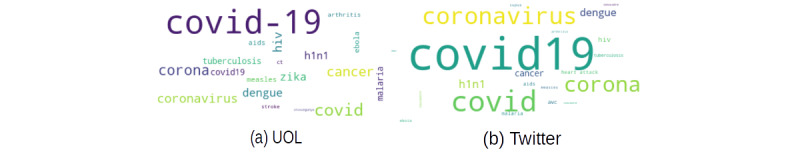
Word clouds showing the most frequent entity mentions in the Disease category: (a) Universo Online; (b) Twitter.

**Figure 12:**
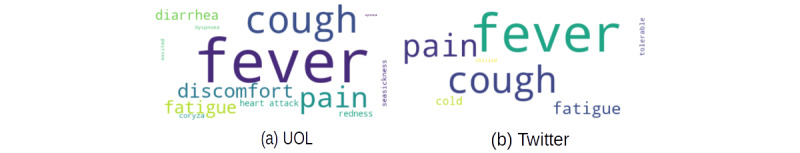
Word clouds showing the most frequent entity mentions in the Symptoms category: (a) Universo Online; (b) Twitter.

**Figure 13:**
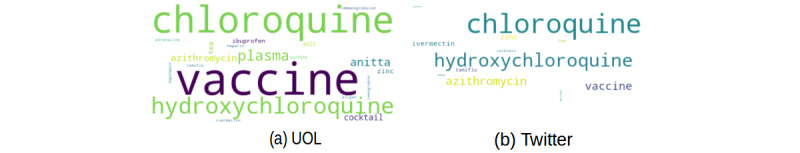
Word clouds showing the most frequent entity mentions in the Drugs category: (a) Universo Online; (b) Twitter.

According to the NER analysis method described in the last section, we compared the main mentions for each entity using word clouds, as this popular text analysis tool provides a visualization of word frequency in a source text while giving more prominence to words that occur more often. To facilitate the understanding of the most representative words by entity, we decided to show the 20 most frequently mentioned words in each entity. Words that were incorrectly extracted as belonging to an entity were manually removed. We assessed that our NER analysis method had an average accuracy of approximately 85% among the 20 most frequently mentioned terms. In Figure 9, we present the most frequent person entity mentions for the UOL and Twitter data collections. The entities of Bolsonaro (the president of Brazil) and Trump (the president of the United States) are the most frequently mentioned persons in both collections. Other frequently mentioned persons in both collections are governors and mayors (eg, Doria and Crivella) and other political personalities (eg, Maia and Moro). Figure 10 presents the most frequent organizations (ORG), and it can be observed that the State Department and Federal Department are the most commonly mentioned entities, followed by media companies. It is interesting to note that social media posts refer frequently to formal media (eg, Globo, which is the main television network in Brazil), and UOL news refers frequently to social networks (eg, Instagram and Twitter). Regarding the Disease entity (DIS), we can see in Figure 11 that the main terms are COVID and coronavirus, as expected, and the terms cancer and Dengue (a common tropical disease) are representative. In Figure 12, the Symptoms (SYMP) entity shows the most common COVID-19 symptom terms, namely *pain*, *fever*, and *cough*, as expected. Finally, Figure 13 shows that the Drugs entity (DRUG) is very polarized to the *chloroquine* discussion in both collections; however, UOL media seems to contain more information about vaccines.

**Figure 14:**
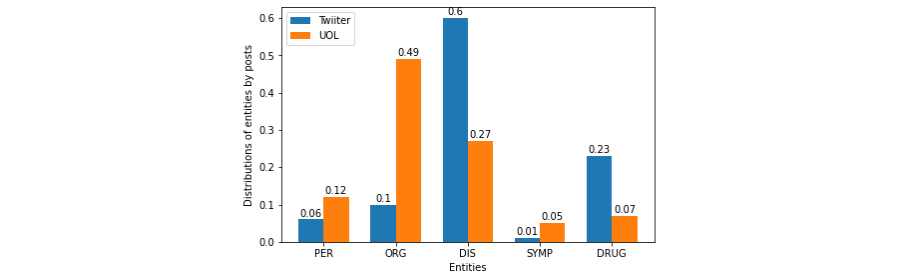
Entity distributions by data set. DIS: Disease; DRUG: Drugs; ORG: Organization; PER: Person; SYMP: Symptoms.

From the word clouds for all these entities, it is important to mention that the found terms are very coherent with their respective entity categories. This fact reinforces that the adopted NER method is valid for the Portuguese language and that this study reflects the Brazilian perception of the COVID-19 pandemic. By comparing both formal and social media, it can be noted that there is no substantial difference regarding the main terms. However, people’s discussions on Twitter have much sparser terms than those on UOL, while the terms in latter seem to be more diverse. Another important difference between the collections can be seen in the entity distribution graph in Figure 14. Both the UOL and Twitter texts obviously contain the main Disease terms (DIS) frequently, with a higher proportion in the size-limited Twitter posts. UOL news articles refer more to official sources of information (ORG entities), while people on Twitter talk more about drug treatments (DRUG). In fact, the administration of chloroquine was the cause of polemic and controversial debate in Brazil, with high politicization [51].

### Topic and Sentiment Changes

Once topics were obtained for all posts in a collection, we classified every document by its topic with highest probability and applied the previously described sentiment analysis. We then grouped all posts by weekly intervals of time, summing the number of documents in each theme and calculating the sentiment averages.

**Figure 15:**
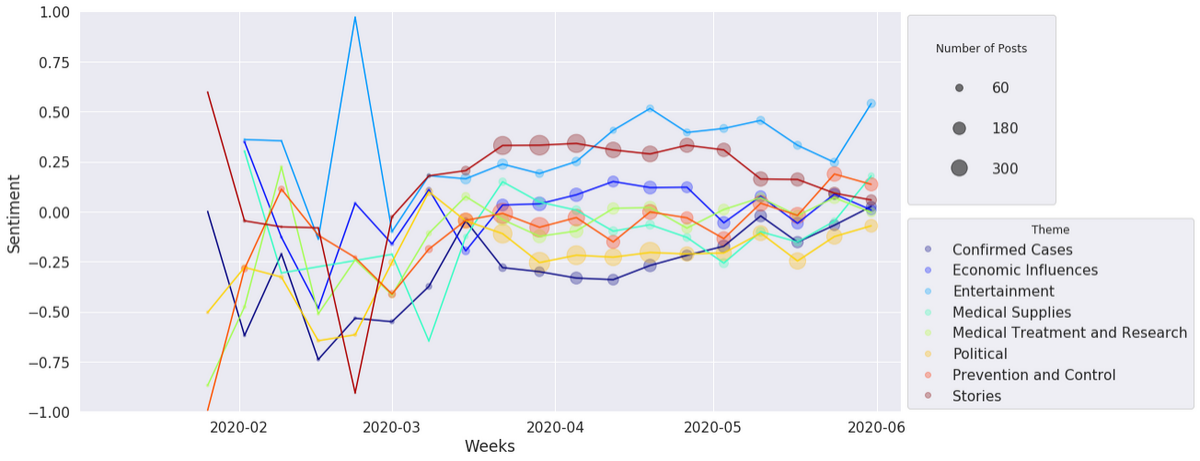
Universo Online sentiment analysis over time.

**Figure 16:**
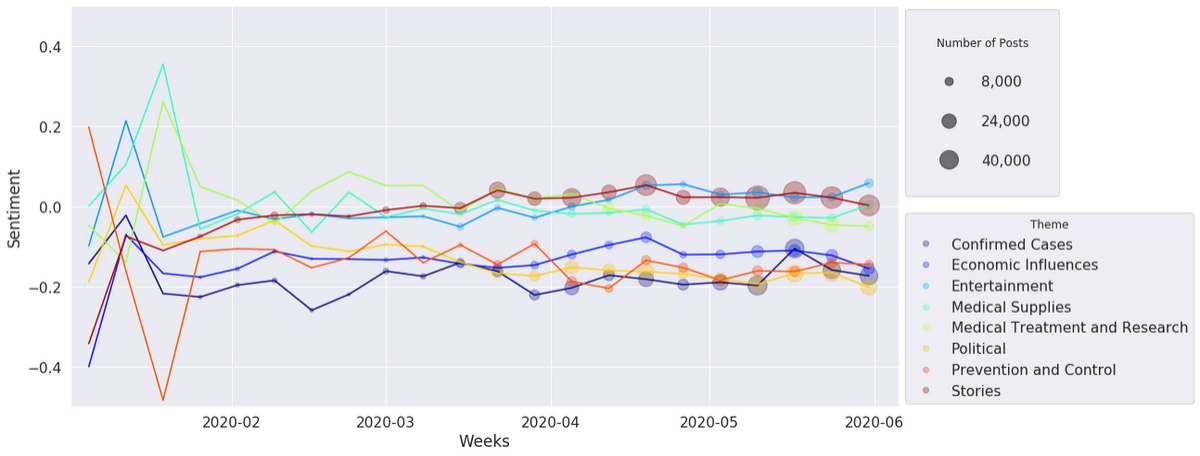
Twitter sentiment analysis over time.

Figure 15 and Figure 16 show how UOL and Twitter sentiments changed over time according to the defined themes. The y-axis presents the sentiment mean for all documents in a given theme, the x-axis presents its evolution grouped by week of the year, and the dot size is proportional to the number of documents for a given theme and week. In both figures, we can see that the number of posts related to COVID-19 was very small during the initial weeks of the pandemic, and the posts presented high sentiment variation due to the impact of some specific posts. The number of posts began to grow considerably after mid-March, when Brazil registered its first death from COVID-19 (March 12, 2020). From the UOL sentiment analysis shown in Figure 15, we can see that all themes are more distributed around the neutral polarity (0.0). The themes of Entertainment and Stories have more positive averages (around 0.25), while Confirmed Cases (involving the number of cases and deaths) and Political are more negative themes (–0.25). Confirmed Cases reached a minimum representative polarity point (with more than 180 posts) by mid-April, just when the curves of confirmed cases and deaths started to scale exponentially. By the end of May, several themes presented a sentiment improvement; this coincides with the plateau of cases and deaths in several Brazilian capitals, such as Manaus, São Paulo, and Rio de Janeiro. For the Twitter collection, as shown in Figure 16, all the themes are positioned lower on the sentiment scale. Political, Confirmed Cases, Prevention and Control, and Economic Influences are more negative (near –0.2), while other themes are close to neutral polarity (0.0). For Twitter posts, it is possible to see that Economic Influences and Prevention and Control are positioned lower on the sentiment scale than UOL news. In fact, much discussion occurred regarding the need for quarantine or social distancing and the impact of these measures on the unemployment rate. This finding is reinforced by the observation that the Economic Influences sentiment increased on average in the beginning of April, when the government announced financial aid for autonomous workers [52]. Unlike UOL articles, Twitter had not yet shown any positivity by the end of May, and Economic Influences showed an additional decrease. We evaluated the standard deviations of the sentiment means, and we noted that they did not change greatly over time or among the themes; therefore, we omitted these data from the graphs. However, slight differences were observed between news (SD ~0.7) and social media (SD ~0.5). These standard deviations show that both sources present a high diversity of sentiments.

## Discussion

### Principal Results

From a general point of view, we can observe that UOL articles and Twitter posts were concerned about the same main COVID-19 topics and themes. For instance, the topics and themes were very similar for both types of media, and this was reflected in the most common entity mentions. This study suggests that formal news media and social media influence each other; we found a representative cross-reference in the Organization entity graph.

The main differences found between UOL and Twitter pertain to the distribution of the main themes, diversity of entities, and overall sentiment about subjects related to COVID-19. Formal media naturally refers more to official entities and their recommendations. This can be seen in its top themes (Political and Prevention and Control), top entity groups (Organization and Disease), and diversity of entity mentions. Twitter, in contrast, is very focused on personal opinions and cases, as demonstrated by its top theme (Stories) and entity groups (Disease and Drugs). Additionally, social media tended to have a more negative polarity for all themes, while formal media seemed to present almost neutral polarity on average. Together with the very high number of collected tweets during the period, which shows that discussion about the disease was very active, we can observe the severity of the pandemic in Brazil and people’s concerns about it.

It is remarkable how the subject of COVID-19 was the target of political polarization in Brazil. This theme was frequently discussed on both formal and social media, with higher negative sentiments over time. Drugs was the second most common entity in social media discussions, and it was very focused on the use of chloroquine to treat patients with COVID-19. A suggested hypothesis to explain this finding is that Brazil's government stated many times that this drug could help treat COVID-19 while minimizing the severity of the disease. In fact, in all the periods examined in this research, the government and formal media positioned themselves in opposite fields in this discussion, which is reflected in the high number of citations to political organization entities and in the disproportional reference to this specific drug.

Finally, by applying the proposed methodology, it was possible to observe the main information being conveyed and how people were reacting to it. This provides a way to monitor the evolution of a pandemic and its effects. Moreover, we believe this information can be useful for researchers and authorities to identify potentially controversial aspects, address possible misinformation, and establish better public policies for action and communication with the population.

### Limitations

We discuss some limitations that can be attributed to this study as follows.

We retrieved data using a set of keywords; therefore, our data may have excluded tweets from users who wrote about the COVID-19 pandemic using different target keywords. A further limitation is that Twitter and UOL do not publish data about the profiles of their users, such as age, gender, or social class. Therefore, it was not possible to perform a stratified analysis of the users, and the results thus may not reflect the entire Brazilian population. A possible hypothesis is that different media reach different segments of society (eg, news media sites are accessed more frequently by more educated people); therefore, these differences may be reflected in the discovered topic distributions and sentiments. Thus, our findings may not be generalizable to other social media platforms or other communication media, such as television or radio. Moreover, the presented results for the selected vehicles may present some bias. For instance, a specific news media source may present a political leaning that can affect the sentiment about some themes. Therefore, while it is not our focus to explore possible bias and its impact on the results, caution is advised before assuming their generalization.

### Conclusions

People rely on data published on the web to better understand recent global crises, and this is also occurring during the COVID-19 pandemic. News media web sites and social media are two distinguished channels of timely information. In this paper, we have proposed a methodological approach to analyze this type of media and to answer some questions regarding the COVID-19 pandemic in Brazil. The results presented and discussed in this study are particularly important because they

make it possible to understand the difference between two data sources in how they cover global crises. In addition, this paper provides a method that uses several computational techniques to process textual social media in a language other than English. As the main contribution, this method resulted in observations that can aid understanding of the COVID-19 pandemic, with a better and more meaningful sentiment timeline.

In future work, we intend to extend this study to include data from longer periods of time, even after the pandemic ends. The idea is to understand how existing media platforms and people will react when they return to a normal situation and whether some trauma will remain. Additionally, we think that the proposed methodology is useful for studying other events of interest, such as other catastrophes and elections. Therefore, we intend to improve it by implementing a tool and applying it to new study cases.

## Abbreviations

LDA: latent Dirichlet allocation
MALLET: Machine Learning for Language Toolkit
NER: named entity recognition
SARS: severe acute respiratory syndrome
UOL: Universo Online
VADER: Valence Aware Dictionary and Sentiment Reasoner
